# Federal inspection timing, not compliance, associated with nursing home post-disaster outcomes

**DOI:** 10.1093/haschl/qxaf244

**Published:** 2025-12-19

**Authors:** Natalia Festa, Kelsey Alexovitz, Manali Phadke, John O’Leary, Kendra Davis-Plourde, Emma Zang, Kai Chen, Jill Kelly, David M Dosa, Andrew Cohen, Thomas M Gill

**Affiliations:** Department of Internal Medicine, Yale School of Medicine, New Haven, CT 06510, United States; VA Connecticut Healthcare System, West Haven, CT 06516, United States; Department of Internal Medicine, Yale School of Medicine, New Haven, CT 06510, United States; VA Connecticut Healthcare System, West Haven, CT 06516, United States; VA Connecticut Healthcare System, West Haven, CT 06516, United States; Department of Biostatistics, Yale School of Public Health, New Haven, CT 06510, United States; Department of Internal Medicine, Yale School of Medicine, New Haven, CT 06510, United States; VA Connecticut Healthcare System, West Haven, CT 06516, United States; Department of Biostatistics, Yale School of Public Health, New Haven, CT 06510, United States; Department of Sociology, Yale University, New Haven, CT 06510, United States; Department of Environmental Health Sciences, Yale School of Public Health, New Haven, CT 06510, United States; Department of Environmental Health Sciences, Yale School of Public Health, New Haven, CT 06510, United States; Department of Medicine, Division of Geriatrics, University of Massachusetts Medical School, Worcester, MA 01655, United States; Department of Internal Medicine, Yale School of Medicine, New Haven, CT 06510, United States; VA Connecticut Healthcare System, West Haven, CT 06516, United States; Department of Internal Medicine, Yale School of Medicine, New Haven, CT 06510, United States

**Keywords:** CMS, long-term care, geriatrics, regulation, compliance

## Introduction

In response to earlier disasters that exposed critical deficits in nursing home preparedness, the Centers for Medicare & Medicaid Services (CMS) expanded its regulatory oversight by incorporating explicit emergency preparedness standards into the existing Life Safety Code (LSC) survey in 2016.^1,2^ Despite federal efforts to strengthen nursing home emergency preparedness, multiple audits indicate that facilities often achieve only superficial administrative compliance, which does not translate into effective disaster response.^3,4^ This disconnect merits critical examination, given that the operational investments required to achieve compliance may divert resources from direct patient care.^5^ Moreover, the empirical basis for the current LSC framework is limited, raising fundamental uncertainties as to its effectiveness in improving post-disaster outcomes for nursing home residents.

This retrospective cohort study examined the influence of LSC survey timeliness and compliance status on post-disaster outcomes among nursing home residents exposed to Hurricane Ian (September 2022).

## Data and methods

We identified Medicare beneficiaries aged ≥65 years residing in nursing homes exposed to Hurricane Ian (September 2022). We classified nursing homes as exposed to Hurricane Ian if their geocoded locations fell within its wind swath, as designated by the National Hurricane Center (NHC), and differentiated exposure intensity using pre-established thresholds from the NHC.^6^

The outcomes of interest included all-cause 30-day mortality (primary) and hospitalization (secondary).

We operationalized two explanatory variables using LSC survey data for each nursing home in the 16-months preceding Hurricane Ian, corresponding to the maximum recommended interval between surveys.^7,8^ First, we identified facilities with lapsed inspections, defined as those lacking any documented LSC survey within the 16-month window. Second, among facilities with completed inspections, we classified facilities as noncompliant if they were assigned one or more deficiencies. We provide additional details in the Appendix.

### Statistical analysis

We employed inverse probability of treatment weighting with stabilized propensity scores to balance resident, facility, and area-level characteristics that could confound the association between regulatory oversight and post-disaster outcomes (Appendix). We estimated separate marginal Cox proportional hazards models for each explanatory variable and outcome. We report hazard ratios (HR) with 95% confidence intervals (95% CI).

## Results

The 920 nursing homes exposed to Hurricane Ian housed 56 825 residents with a mean age of 81.1 years [standard deviation (SD) 9.1] (Table AS1). Most residents were female (65.4%) and received long-term care (84.5%), while 72.5% identified as Non-Hispanic White. Of the 920 nursing homes, 209 (22.7%) had lapsed inspections (Table AS2). Of the remaining 711 facilities with documented inspections, 309 (43.5%) were noncompliant with at least one LSC standard.

**Figure 1.**
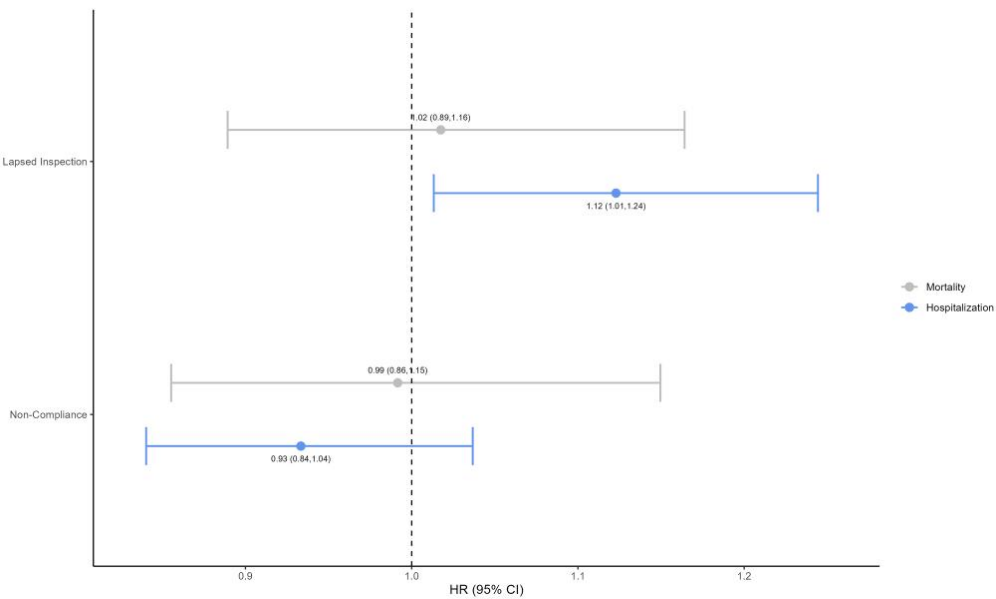
The effect of lapsed inspection and LSC noncompliance on adverse post-disaster outcomes for nursing home residents. We report hazard ratios from Cox proportional hazards models after balancing sample characteristics using inverse probability of treatment weighting. The average effective sample size for residents in lapsed facilities was 12 243 and 42 876 for nonlapsed facilities. The average effective sample size for residents in noncompliant facilities was 15 306 and 22 010 for compliant facilities. LSC, life safety code; CI, confidence interval.

Among all facilities, lapsed inspections were associated with a 12% increase in the risk of post-disaster hospitalization (HR 1.12, 95% CI 1.01-1.24), but were not associated with post-disaster mortality (HR 1.02, 95% CI 0.89-1.16) (Figure 1). In contrast, noncompliance among facilities with completed inspections demonstrated no association for either post-disaster mortality (HR 0.99, 95% CI 0.86-1.15) or hospitalization (HR 0.93, 95% CI 0.84-1.04).

In sensitivity analyses, our results were robust to alternative assumptions (Tables SA3–SA5).

## Discussion

This study examined whether the timeliness of CMS LSC inspections and nursing home compliance with LSC standards were associated with adverse post-disaster outcomes among nursing home residents exposed to Hurricane Ian. We found that residents of facilities with lapsed inspections experienced a greater risk of post-disaster hospitalization but not mortality, while compliance status among inspected facilities was not associated with either post-disaster mortality or hospitalization. These findings suggest that the inspection process itself, rather than compliance status, may constitute the primary mechanism through which regulatory oversight influences residents’ post-disaster outcomes.

The protective association between inspection timeliness and reduced post-disaster hospitalizations indicates that regular surveyor visits may motivate improvements to emergency preparedness practices beyond those captured by LSC standards. This finding aligns with evidence from routine (nondisaster) care settings indicating that longer intervals between CMS Standard (Health) inspections are associated with poorer care quality.^9^ There are at least two potential explanations for the observed associations. First, the anticipation of inspections may prompt facilities to maintain preparedness systems and protocols at levels exceeding minimum regulatory requirements. Second, surveyors possessing specialized expertise in emergency management may offer informal guidance that enables nursing home staff to strengthen their disaster-preparedness procedures. The observed association between inspection timeliness and hospitalization but not mortality may reflect temporal patterns, with hospitalization representing a more proximal outcome. This pattern is consistent with evidence that disaster-related disruptions to healthcare access have emerged as a principal mechanism of harm for populations with complex clinical and functional needs, such as nursing home residents.^10^

The absence of associations between compliance status and post-disaster outcomes may be explained by two principal mechanisms. First, surveyors near-uniformly grade LSC deficiencies as lower scope and severity than deficiencies documented during Standard (Health) Inspections, despite applying the same CMS scale designed to calibrate enforcement actions for instances of noncompliance.^11^ This results in both minimal and undifferentiated enforcement actions for distinct deficiencies and may, therefore, fail to signal which remedial actions should be prioritized. Second, current, dichotomous LSC standards cannot distinguish between facilities meeting minimum requirements and those implementing comprehensive preparedness approaches.

The following limitations should be noted. First, this study focused on a single, recent event (Hurricane Ian). Although the effects of noncompliance with federal emergency preparedness standards may vary across disaster categories, hurricanes remain among the most frequent and predictable disasters to which coastal nursing homes are exposed. Therefore, our findings provide foundational evidence regarding the potential efficacy of federal emergency preparedness oversight in a salient real-world disaster scenario. Second, the observational design precludes causal attribution regarding the effects of inspection timeliness. Third, data regarding surveyor expertise or experience were unavailable, preventing examination of whether the effects of inspections vary based on surveyor qualifications. Fourth, our use of administrative data prevented assessment of specific disaster-response activities that may mediate relationships between regulatory oversight and post-disaster outcomes.

The results of this study suggest that regular inspections may influence preparedness through organizational vigilance and informal educational exchange rather than through formal compliance determinations. Amid ongoing reassessment of federal disaster-response policy, deliberations concerning the Federal Emergency Management Agency (FEMA) indicate that response efforts may increasingly fall under state and local purview.^12^ Under such conditions, organizational preparedness within nursing homes would assume heightened importance as a determinant of resident outcomes. Future federal efforts to prevent adverse post-disaster outcomes should consider both the timeliness of regulatory oversight and the development of assessment methods that better capture variation in the quality of nursing home preparedness.

## Contribution statement

N.F. is the guarantor of this research. N.F. and K.A. had full access to the data and take responsibility for its integrity and the accuracy of the analysis. Concept and Design: N.F., K.A., D.D., and T.G. Acquisition, analysis, or interpretation of data: N.F., K.A., M.P., J.O., and K.D.P. Drafting of the manuscript: All. Critical revision of the manuscript for intellectual content: All. Statistical Analysis: N.F. and K.A. Obtained funding: N.F. Administrative, technical, or material support: K.D.P., J.K., E.Z., K.C., D.D., A.C., and T.G. Supervision: K.D.P., J.K., E.Z., K.C., D.D., A.C., and T.G.

## Supplementary Material

qxaf244_Supplementary_Data
