# Supplementary material for: Federal inspection timing, not compliance, associated with nursing home post-disaster outcomes
Source: Health Aff Sch. 2025 Dec 19;4(1):qxaf244. doi: 10.1093/haschl/qxaf244 (PMC12778326; doi:10.1093/haschl/qxaf244)
Supplement: qxaf244_Supplementary_Data [file qxaf244_supplementary_data.zip › Appendix.docx]

**Appendix**

**A. Data and Methods**

This retrospective cohort study was approved by both the Yale and VA Institutional Review Boards, and was exempt (Category 4) from Human Subjects Review. The study followed the Strengthening the Reporting of Observational Studies in Epidemiology (STROBE) reporting guidelines.

*Data Sources and Cohort Creation*

We used information from the 100% Medicare Beneficiary Summary File (MBSF), Medicare Provider Analysis and Review (MedPAR), and Minimum Data Set (MDS) files to establish the cohort of individuals located within exposed nursing homes on September 25, 2022 (Day 0). We designated Day 0 as 72-hours before landfall in the United States because this corresponds to the timepoint at which facilities implement operational decisions (e.g., evacuation, surge staffing) that are attributable to the anticipated storm.^7^

*Inverse Probability of Treatment Weighting and Covariate Selection*

We employed inverse probability of treatment weighting using generalized linear model stabilized propensity score weights to achieve covariate balance between comparison groups. This approach enables retention of the complete analytic sample through statistical reweighting rather than exclusion of unmatched observations, thereby preserving statistical power while achieving covariate balance.^1,2^ We calculated the effective sample size for each weighted group, which represents the number of independent observations in a hypothetical unweighted sample that would provide equivalent statistical precision.^3–5^ We applied this weighting method for two distinct comparisons to balance characteristics and prepare the data to estimate the average treatment effect. For the lapsed inspection comparison, we weighted residents from facilities with both completed and lapsed inspections. For the non-compliance comparison, we first restricted the sample to facilities with completed inspections and then weighted residents from facilities that were compliant and non-compliant with LSC standards.

We conducted the analysis at the resident level and selected covariates for inclusion in the inverse probability of treatment weighting models based on their potential to confound the associations between regulatory oversight and resident outcomes. We applied multiple imputation by chained equations to impute missing values (<5.8%).^6^ The resident-level characteristics included age, sex, race and ethnicity, long-term care status (skilled nursing stay ≥90 days), and the MDS-CHESS score, a validated composite measure of clinical conditions, cognitive impairment, and functional status.^7,8^ We classified residents with skilled nursing stays ≥90 days as long-term care residents, with the remaining residents classified as post-acute.^9^ For nursing home admissions interrupted by a hospitalization, we used prior MDS assessments to adjudicate long-term care status.^9,10^ We incorporated organizational characteristics of the nursing home where each resident resided, including bed count, for-profit ownership status, and CMS 5-star rating from the CMS Provider Information Files, as well as baseline registered nurse, licensed practical nurse, and certified nurse assistant hours per resident per day from the Payroll Based Journal.^11,12^ We classified hurricane exposure severity for each resident based on the maximum sustained wind speeds at the location of the nursing home where they resided (≥39, ≥58, or ≥74 miles per hour).^13^ We also incorporated area-level characteristics, including the county-level rurality classification from the National Center for Health Statistics and the county-level Social Vulnerability Index from the Centers for Disease Control, as well as state.^14,15^ We assessed covariate balance at the resident level using standardized mean differences before and after applying inverse probability of treatment weights, with balance achieved when standardized differences were ≤0.1. **Figure A1** presents balance for the lapsed inspection analysis among all facilities; **Figure A2** presents balance for the non-compliance analysis among inspected facilities.

*Statistical Analysis*

We estimated marginal Cox proportional hazards models, with facility-robust standard errors, to evaluate associations between each explanatory variable and post-disaster outcomes, as described in the main text.

*Sensitivity Analyses Overview*

We conducted a set of sensitivity analyses to evaluate the robustness of our findings to alternative assumptions. First, we modified our inverse probability of treatment weighting approach, using alternative weighting (entropy balancing), to evaluate if this altered the observed associations between our explanatory variables and adverse post-disaster outcomes. Second, given the zero-inflated and right-skewed distribution of LSC deficiencies (median 0, IQR 0-14), we examined whether facilities with a higher degree of non-compliance incurred an increased likelihood of adverse post-disaster outcomes. To do so, we dichotomized facilities at two alternative thresholds, comparing facilities in the top quartile (>75th percentile) versus those below, and facilities in the top decile (>90th percentile) versus those below. Third, we evaluated the robustness of our findings to an alternative criterion to classify exposure to Hurricane Ian. To do so, we modified the sample inclusion criteria to incorporate only nursing homes located within counties that received Federal Emergency Management Agency (FEMA) Major Disaster Declarations (MDD). As MDD designations are issued based on post-hoc damage assessments, this alternative classification may capture hazardous exposures, such as severe flooding, that are distinct from those captured by wind-swath-based exposure definitions.

**Figure A1: Covariate Balance for Lapsed Inspection Analysis Using Inverse Probability of Treatment Weighting**

**
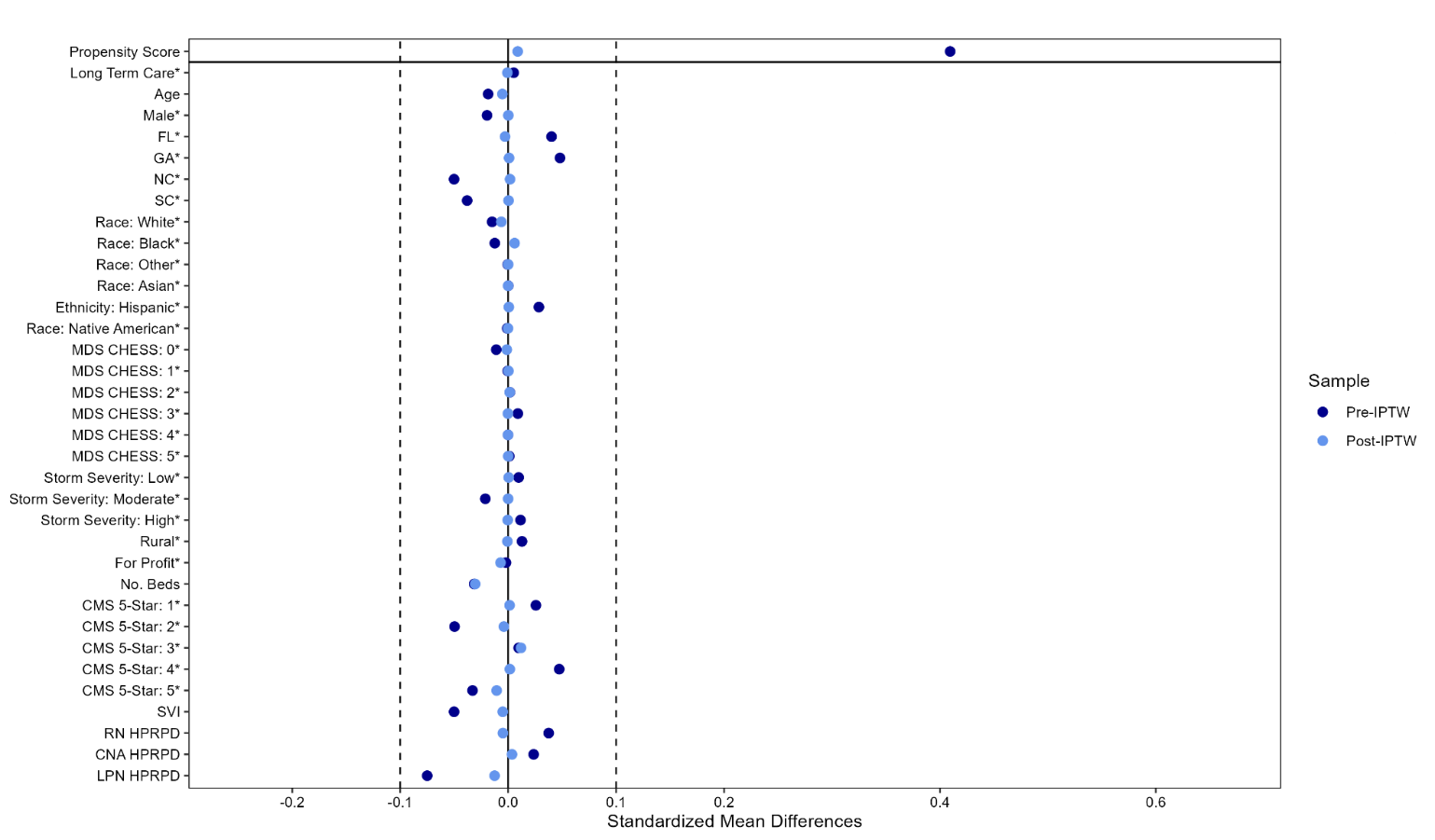
**

**Legend**: We report standardized mean differences for all covariates before and after inverse probability of treatment weighting using generalized linear model propensity score weights; dashed lines indicate SMD thresholds of ±0.1.

**Abbreviation(s)**: SMD: Standardized Mean Difference; IPTW: Inverse Probability of Treatment Weighting

**Figure A2: Covariate Balance for Non-Compliance Analysis Using Inverse Probability of Treatment Weighting**

**
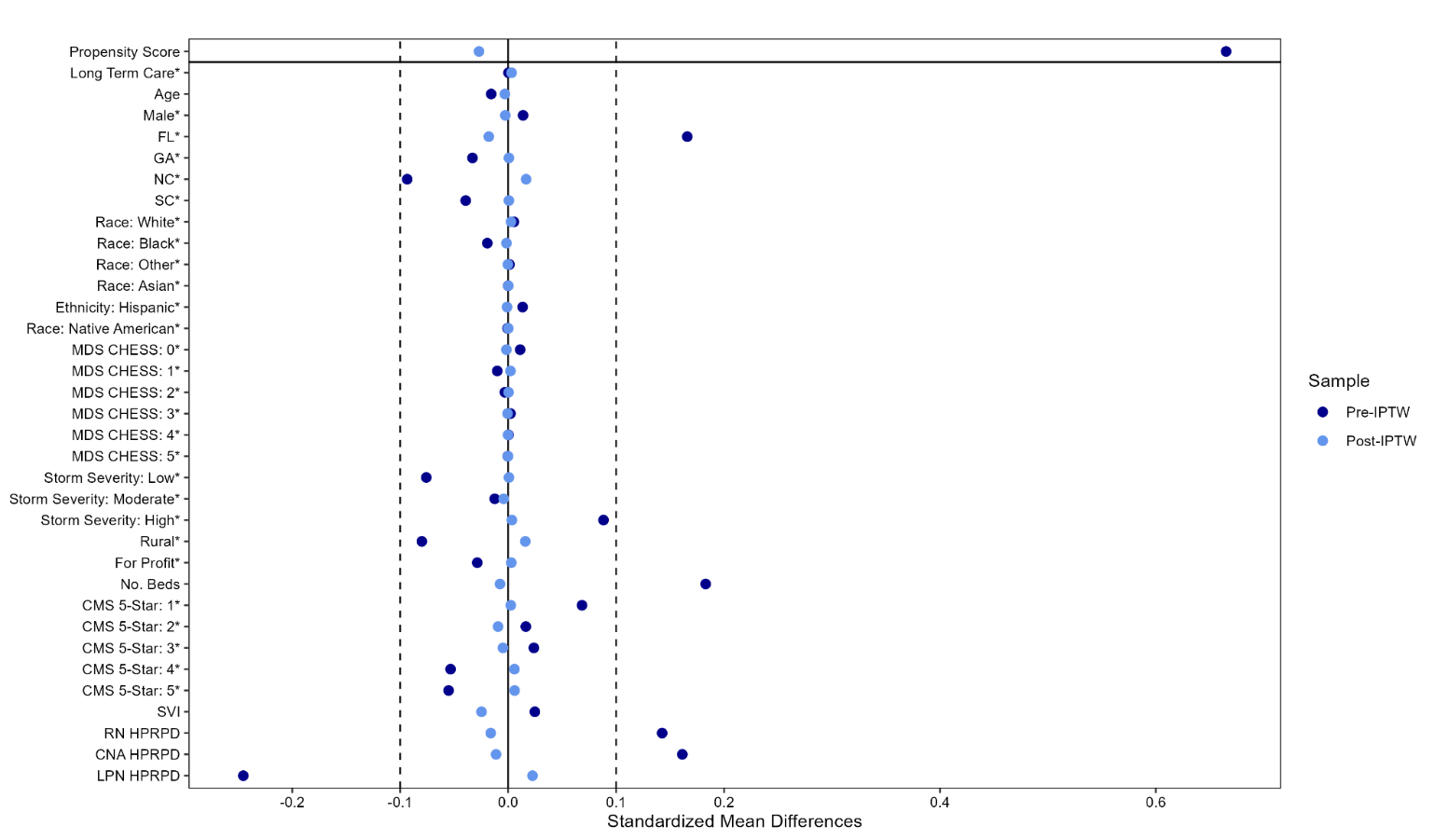
**

**Legend**: We report standardized mean differences for all covariates before and after inverse probability of treatment weighting using generalized linear model propensity score weights; dashed lines indicate SMD thresholds of ±0.1.

**Abbreviation(s)**: SMD: Standardized Mean Difference; IPTW: Inverse Probability of Treatment Weighting

**B. Results of Supplemental Analyses**

**Table A1**: Sample Characteristics

| Descriptive Characteristics^(a)^ | |
| --- | --- |
| No. Residents | 56,825 |
| Resident Census | |
| Average Age (years) | 81.1 (9.1) |
| Female | 37,139 (65.4%) |
| Race |  |
| Asian | 336 (0.6%) |
| Black | 12,561 (22.2%) |
| White | 41,176 (72.5%) |
| Other Identification | 594 (1.0%) |
| Ethnicity |  |
| Hispanic | 2,171 (3.8%) |
| Percent Long-Term Care Residents | 48,019 (84.5%) |
| MDS-CHESS Score | 2.0 [1.0 - 3.0] |
| No. Nursing Homes | 920 |
| Organizational | |
| No. Beds | 116.2 (47.0) |
| Registered Nurse HPRPD^(b)^ | 0.3 [0.2 - 0.5] |
| Licensed Practical Nurse HPRPD^(b)^ | 0.8 [0.7 - 1.0] |
| Certified Nursing Assistant HPRPD^(b)^ | 2.2 [2.0 - 2.4] |
| For-Profit Ownership | 671 (72.9%) |
| CMS 5-Star Rating | 3.0 (1.4) |
| Area | |
| Rural | 132 (14.3%) |
| Social Vulnerability Index^(c)^ | 0.8 [0.6 - 0.8] |
| Storm Severity |  |
| Low | 540 (58.7%) |
| Moderate | 285 (31.0%) |
| High | 95 (10.3%) |
| State |  |
| FL | 634 (68.9%) |
| GA | 54 (5.9%) |
| NC | 141 (15.3%) |
| SC | 91 (9.9%) |

**Footnotes**: (a) We used multiple imputation to impute 10 sets of values for the <5.8% of missing observations. For continuous variables with a normal distribution, values are reported as mean (standard deviation (SD)); values for non‐normal distributions are reported as median [interquartile range]. Dichotomous variables are reported as n (%), and other categorical variables reported as % (standard error (SE)); (b) Average staff hours per resident per day (HPRPD) were derived from the CMS Payroll‐Based Journal for the month preceding Hurricane Ian; (c) The Social Vulnerability Index ranges from 0 to 1, with higher values indicating greater vulnerability

**Abbreviations**: CMS: Centers for Medicare & Medicaid Services; MDS: Minimum Data Set; CHESS: Changes in Health, End-Stage Disease and Signs and Symptoms; HPRPD: hours per resident per day

**Table A2**: Description of Deficiencies and Lapsed Life Safety Code Inspections for Nursing Homes

| No. Nursing Homes(a) | 920 |
| --- | --- |
| Nursing Homes with Lapsed Inspections | |
| Percentage with Lapsed Inspections | 209 (22.7%) |
| Average Duration Lapse (months) | 20.8 (6.5) |
| Median Duration of Lapse (months) | 18.2 [16.9 - 20.2] |
| Deficiencies among Inspected Nursing Homes(b) | |
|  | 711 |
| Facilities with Any Life Safety Code Deficiency | 309 (43.5%) |
| Average Life Safety Code Deficiencies Count | 11.3 (20.5) |
| Median Life Safety Code Deficiencies Count | 0.0 [0.0 - 14.0] |
| Stratified by Deficiency Category | |
| Facilities with Any E-Tag Deficiency(c) | 74 (10.4%) |
| Average E-Tag Deficiencies Count | 1.9 (7.2) |
| Median E-Tag Deficiencies Count | 0.0 [0.0 – 0.0] |
| Facilities with Any K-Tag Deficiency(d) | 289 (40.6%) |
| Average K-Tag Deficiencies Count | 9.4 (17.7) |
| Median K-Tag Deficiencies Count | 0.0 [0.0 – 12.0] |

**Footnotes**: (a) The mean and standard deviation (SD) are reported for continuous variables, number and percent are reported for categorical variables; (b) Percentages are calculated as percent of facilities that did not lapse in their Life Safety Code Inspection (n=711) (c) E-tag deficiencies correspond to emergency preparedness standards for CMS-certified nursing homes; (d) K-tag deficiencies correspond to fire preparedness and building code standards for CMS-certified nursing homes

**Abbreviations**: CMS: Centers for Medicare & Medicaid Services

**Table A3:** The Effect of Lapsed Inspection and LSC Non-Compliance on Adverse Post-Disaster Outcomes for Nursing Home Residents, under Alternative IPTW Approach

| **Post-Disaster Outcome** | Lapsed Inspection^(a)^  Hazard Ratio (95% CI) | Non-Compliance^(b)^  Hazard Ratio (95% CI) |
| --- | --- | --- |
| 30-Day Mortality | 1.02 (0.89,1.17) | 0.99 (0.86,1.14) |
| 30-Day Hospitalization | 1.13 (1.02,1.25)* | 0.94 (0.85,1.04) |

**Footnotes**: (a) The effective sample size for residents in lapsed facilities was 12,012 and 42,937 for non-lapsed facilities; (b) The effective sample size for residents in non-compliant facilities was 16,814 and 21,938 for compliant facilities.

**Abbreviation(s)**: IPTW: Inverse Probability of Treatment Weighting; LSC: Life Safety Code; CI: Confidence Interval

**Table A4:** The Effect of the Number of Deficiencies on Adverse Post-Disaster Outcomes for Nursing Home Residents

| **Post-Disaster Outcome** | Number of Deficiencies  75^th^ Percentile^(a)^  Hazard Ratio (95% CI) | Number of Deficiencies  90^th^ Percentile^(b)^  Hazard Ratio (95% CI) |
| --- | --- | --- |
| 30-Day Mortality | 0.95 (0.80,1.13) | 1.09 (0.82,1.45) |
| 30-Day Hospitalization | 1.07 (0.96,1.20) | 1.10 (0.93,1.29) |

**Footnotes**: (a) The 75^th^ percentile is 14 deficiencies among facilities that underwent Life Safety Code Inspection (n=711). The average effective sample size for residents in facilities with >75^th^ percentile of deficiencies was 6,851 and 30,970 for ≤75^th^ percentile of deficiencies. (b) The 90^th^ percentile is 39 deficiencies among facilities that underwent Life Safety Code Inspection (n=711). The average effective sample size for residents in facilities with >90^th^ percentile of deficiencies was 2,493 and 38,878 for ≤90^th^ percentile of deficiencies.

**Abbreviation(s)**: LSC: Life Safety Code; CI: Confidence Interval

**Table A5:** The Effect of Lapsed Inspection and LSC Non-Compliance on Adverse Post-Disaster Outcomes for Nursing Home Residents, using Alternative Sample Inclusion Criterion^(a)^

| **Post-Disaster Outcome** | Lapsed Inspection^(b)^  Hazard Ratio (95% CI) | Non-Compliance^(c)^  Hazard Ratio (95% CI) |
| --- | --- | --- |
| 30-Day Mortality | 1.17 (0.98, 1.38) | 1.05 (0.89, 1.24) |
| 30-Day Hospitalization | 1.19 (1.03, 1.36)* | 0.97 (0.85, 1.11) |

**Footnotes**: (a)We modified the sample inclusion criteria to incorporate only nursing homes within counties that received a FEMA Major Disaster Declaration; (b) The average effective sample size for residents in lapsed facilities was 6,094 and 23,897 for non-lapsed facilities; (c) The average effective sample size for residents in non-compliant facilities was 11,545 and 10,564 for compliant facilities.

**Abbreviation(s)**: FEMA: Federal Emergency Management Agency; LSC: Life Safety Code; CI: Confidence Interval

**Appendix References**

1. Austin PC, Stuart EA. Moving towards best practice when using inverse probability of treatment weighting (IPTW) using the propensity score to estimate causal treatment effects in observational studies. *Stat Med*. 2015;34(28):3661. doi:10.1002/SIM.6607

2. Austin PC. The use of propensity score methods with survival or time-to-event outcomes: reporting measures of effect similar to those used in randomized experiments. *Stat Med*. 2014;33(7):1242-1258. doi:10.1002/SIM.5984

3. Zakrison TL, Austin PC, McCredie VA. A systematic review of propensity score methods in the acute care surgery literature: avoiding the pitfalls and proposing a set of reporting guidelines. *Eur J Trauma Emerg Surg*. 2018;44(3):385-395. doi:10.1007/S00068-017-0786-6

4. Arguelles GR, Shin M, Lebrun DG, DeFrancesco CJ, Fabricant PD, Baldwin KD. A Systematic Review of Propensity Score Matching in the Orthopedic Literature. *HSS J*. 2022;18(4). doi:10.1177/15563316221082632

5. Austin PC. Informing power and sample size calculations when using inverse probability of treatment weighting using the propensity score. *Stat Med*. 2021;40(27):6150. doi:10.1002/SIM.9176

6. van Buuren S, Groothuis-Oudshoorn K. mice: Multivariate Imputation by Chained Equations in R. *J Stat Softw*. 2011;45(3):1-67. doi:10.18637/JSS.V045.I03

7. Hirdes JP, Frijters DH, Teare GF. The MDS-CHESS scale: a new measure to predict mortality in institutionalized older people. *J Am Geriatr Soc*. 2003;51(1):96-100. doi:10.1034/J.1601-5215.2002.51017.X

8. Ogarek JA, McCreedy EM, Thomas KS, Teno JM, Gozalo PL. Minimum Data Set Changes in Health, End-Stage Disease and Symptoms and Signs Scale: A Revised Measure to Predict Mortality in Nursing Home Residents. *J Am Geriatr Soc*. 2018;66(5):976-981. doi:10.1111/JGS.15305

9. Intrator O, Grabowski DC, Zinn J, et al. Hospitalization of Nursing Home Residents: The Effects of States’ Medicaid Payment and Bed-Hold Policies. *Health Serv Res*. 2007;42(4):1651. doi:10.1111/J.1475-6773.2006.00670.X

10. Goodwin JS, Li S, Zhou J, Graham JE, Karmarkar A, Ottenbacher K. Comparison of methods to identify long term care nursing home residence with administrative data. *BMC Health Serv Res*. 2017;17(1). doi:10.1186/S12913-017-2318-9

11. Centers for Medicare & Medicaid Services. Staffing Data Submission Payroll Based Journal (PBJ). Published 2022. Accessed June 1, 2022. https://www.cms.gov/Medicare/Quality-Initiatives-Patient-Assessment-Instruments/NursingHomeQualityInits/Staffing-Data-Submission-PBJ

12. Centers for Medicare and Medicaid Services. Provider Information. Published online 2024. https://data.cms.gov/provider-data/dataset/4pq5-n9py

13. Bucci L, Alaka L, Hagen A, Delgado S, Beven J. *National Hurricane Center Tropical Cyclone Report: Hurricane Ian (AL092022)*.; 2023. https://www.nhc.noaa.gov/data/tcr/AL092022_Ian.pdf

14. Ingram DD, Franco SJ. NCHS urban-rural classification scheme for counties. *Vital Health Stat 2*. 2012;(154):1-65. Accessed December 27, 2023. https://europepmc.org/article/med/22783637

15. Rickless DS, Wilt GE, Sharpe JD, Molinari N, Stephens W, Leblanc TT. Social Vulnerability and Access of Local Medical Care During Hurricane Harvey: A Spatial Analysis. *Disaster Med Public Health Prep*. Published online 2021. doi:10.1017/DMP.2020.421
